# Use of Oxycodone in Pain Management

**DOI:** 10.5812/aapm.4529

**Published:** 2012-04-01

**Authors:** Mohammad Moradi, Sara Esmaeili, Saeed Shoar, Saeid Safari

**Affiliations:** 1 Development Association of Clinical Studies (DACS), Tehran University of Medical Sciences (TUMS), Tehran, Iran; 2 Students’ Scientific Research Center (SSRC), Tehran University of Medical Sciences (TUMS), Tehran, Iran; 3 Department of Anesthesiology, Rasoul Akram Medical Center, Tehran university of Medical Sciences (TUMS), Tehran, Iran

**Keywords:** Oxycodone, Pain, Analgesics, Opioid

## Abstract

Oxycodone is widely used to alleviate moderate-to severe acute pain, It is an effective analgesic for many types of pain, and is especially useful for paroxysmal spontaneous pain, steady pain, allodynia associated with postherpetic neuralgia, and it is also increasingly used in the management of cancer-related and chronic pain, oxycodone has been found to improve the quality of life of patients with many types of pain.

In 2011, following chemical and physical manipulation, an extended-release form of oxycodone was developed in order to maintain its rate-controlling mechanism. This new formulation significantly improved analgesia among patients with moderate-to-severe chronic osteoarthritis pain with an adverse event profile similar to that of other opioids. The long-term safety and efficacy of extended-release form of oxycodone in relieving moderate-to-severe chronic pain has been demonstrated. In this study we discussed about different aspects of this drug in managing of various types of pain.

Oxycodone is an analgesic opioid medication that is generally used for the relief of moderate-to-severe pain. It was first developed by German researchers in 1916 from opium-derived thebaine (1). Oxycontin was approved by the FDA in 1995, and was introduced into the United States market in 1996 (2). By 2001 it was the best-selling narcotic pain reliever in the United States, and in 2008 sales in the United States reached $2.5 billion (3).

Oxycodone is metabolized by the cytochrome p450 enzyme system in the liver. Only 10% is excreted unchanged in urine (4, 5). Because it is metabolized by CYP3A4 and CYP2D6 enzymes, oxycodone is prone to pharmacokinetic drug interactions (6). Such drug interactions may unexpectedly increase exposure to oxycodone, and lead to potentially dangerous adverse effects such as respiratory depression(7).

Oxycodone is widely used to alleviate moderate-to-severe acute pain, but it is also increasingly used in the management of cancer-related and chronic pain (8, 9). Oxycodone has been found to improve the quality of life of patients with many types of pain (10). Other pain relievers are morphine, heroin, and cocaine (11). The opioids morphine and oxycodone are potent analgesics that are available as extended-release and immediate-release tablets. The indications are the same for both drugs, that is, they are used to treat severe acute and chronic pain (non-malignant or malignant). Few clinical studies have compared morphine and oxycodone directly, and there is no evidence to support one being superior to the other (12). There is also no evidence of a significant difference in analgesia or in the incidence of adverse effects between oxycodone and morphine or hydromorphone. Oxycodone can thus be recommended as an alternative to morphine or hydromorphone for cancer-related pain (13). However, we now know that morphine and oxycodone exert different effects in the sensitized pain system as oxycodone has a greater analgesic effect against skin, muscle, and oesophageal pain. Clinical experience of oxycodone use also indicates that it is superior to morphine in the treatment of some pain conditions (14). Since oxycodone is safer than morphine, it has been used for refractory bone pain, which has a complicated pathophysiological mechanism. Given these characteristics, oxycodone might be a suitable candidate for first-line management of cancer-related pain despite the wide variety of pathophysiologies of such pain (15). A new study has recently reported on 5 clinical cases where oxycodone was effective against pain induced by anti-cancer agents during adjuvant therapy. Pain intensity as measured by a numerical rating scale was decreased to less than 3 out of 10 compared to baseline in every patient but one (16). In a previous experimental pain study in healthy volunteers, morphine and oxycodone were found to have comparable analgesic potency in modulating skin and muscle pain, but oxycodone showed greater analgesic potency for visceral pain (17). Subsequently, another experimental pain study was performed in patients with chronic pancreatitis, and oxycodone was found to be more potent than morphine in attenuating experimental skin, muscle, and visceral pain (18). This supports the theory of different analgesic potencies of morphine and oxycodone when hyperalgesia is present (14). A study in 2011 also showed that a fixed-ratio morphine-oxycodone combination (MoxDuo) produced superior analgesic affects compared with each component individually, but comparable efficacy compared with morphine-equivalent doses in moderate-to-severe postoperative pain (19).

When using opioids, whether initiating therapy or changing from another opioid, it is usually necessary to titrate the dose in order to optimally balance analgesia and side effects because of variability in opioid response both between patients and within the same patient. In 2011, following chemical and physical manipulation, an extended-release form of oxycodone was developed in order to maintain its rate-controlling mechanism. This new formulation was named Remoxy® (King Pharmaceuticals, Inc., Bristol, TN, which was acquired by Pfizer Inc in March 2011) (20). Remoxy significantly improved analgesia among patients with moderate-to-severe chronic osteoarthritis pain with an adverse event profile similar to that of other opioids (21). The long-term safety and efficacy of Remoxy in relieving moderate-to-severe chronic pain has been demonstrated (20). The oxycodone-paired stimulus maintained an operant response, but this effect was dependent on the number of conditioning sessions and on the conditioning dose (22). Oxycodone has also been combined and tested with other drugs in order to determine whether better pain relief can be achieved with less adverse effects. Another study has shown that an oxycodone/naloxone combination (ratio 2:1) provides analgesia with less constipation than other analgesics in non-cancer patients receiving relatively low doses of this formulation (23). Treatment with a combination of carbamazepine, a sodium channel blocker, and oxycodone, a mixed κ- and μ-opioid receptor agonist, may also be useful for alleviating symptoms of trigeminal neuralgia (24). A total of 10 mg of oral oxycodone combined with a low dose of ethanol generated abuse liability-related effects, but when tested separately, they did not. Further psychopharmacological investigations of this combination are warranted in light of these findings as well as the fact that nonmedical use of prescription opioids is sometimes accompanied by the use of ethanol (25). The combination of CR oxycodone + pregabalin could be a valuable long-term therapeutic addition to existing pharmacological options for the treatment of non-cancer pain (26).

A large study evaluating the adverse effects of oxycodone among 601 respondents found that 84.0% experienced side effects with 30.8% being quite or extremely bothered by these effects. A total of 56.2% experienced drowsiness, 53.1% constipation, 43.6% lightheadedness, 42.1% dizziness, 33.1% headache, 31.3% nausea, 27.6% itching, and 14.8% vomiting (27). Generally, oxycodone is better tolerated than morphine (28). Oxycodone also significantly lengthens time estimations relative to placebo. These results suggest that opioids alter temporal processing for intervals greater than 1 s, raising questions about the effect of these drugs on the valuation of future consequences (29). The symptoms of oxycodone withdrawal are the same as those for other opiate-based painkillers, and may include 

“anxiety, nausea, insomnia, muscle pain, muscle weakness, fevers, and other flu-like symptoms”(30). Withdrawal symptoms have also been reported in newborns whose mothers had injected or orally ingested oxycodone during pregnancy (31).
